# Lack of Serological Response by Delivery to Syphilis Treatment Does Not Impact Pregnancy Outcomes

**DOI:** 10.3390/jcm13144031

**Published:** 2024-07-10

**Authors:** Konrad Kaminiów, Agnieszka Kotlarz, Martyna Kiołbasa, Maciej Pastuszczak

**Affiliations:** 1 Clinical Department of Dermatology, Medical University of Silesia, Marii Curie-Skłodowskiej 10, 41-800 Zabrze, Poland; martynakiolbasa@gmail.com (M.K.); maciej.pastuszczak@sum.edu.pl (M.P.); 2 Chair of Gynaecology and Obstetrics, Jagiellonian University Medical College, Mikołaja Kopernika 23, 31-501 Krakow, Poland; a.kotlarz@uj.edu.pl

**Keywords:** syphilis, maternal syphilis, inadequate serological response, pregnancy, neonate

## Abstract

**Objectives:** Maternal syphilis can lead to serious adverse pregnancy outcomes, including neonatal death. A 4-fold decline in blood non-treponemal titer at six months after the treatment of syphilis compared to the baseline is considered as an adequate serological response. However, the duration of normal human gestation does not allow the ascertainment of an adequate serological response. **Aim:** The aim of this study was to assess correlations between the lack of a 4-fold decrease in non-treponemal titer by delivery after syphilis treatment and fetal and newborns’ condition and serological outcomes. **Methods:** Fourteen pregnant patients (gestational age 16–22 weeks) diagnosed with early syphilis (secondary or latent) were treated with intramuscular benzathine penicillin and subsequently monitored clinically, serologically, and ultrasonographically at monthly intervals. Based on the non-treponemal test results at delivery, patients were stratified into two groups: those with a 4-fold decline in titers and those without such a decline. All newborns were clinically and serologically assessed for congenital syphilis at birth and then monitored until serological tests became negative. **Results:** Fifty percent of the included women did not achieve a 4-fold decline in non-treponemal titer by delivery. Patients from the group showing a 4-fold decline in RPR titer at delivery and those without such a decline did not differ in basic demographic and clinical characteristics or in ultrasound parameters used for fetal assessment. Based on the clinical and laboratory assessments of newborns on the day of delivery and during a 6-month follow-up, none were diagnosed with congenital syphilis or required treatment for syphilis. **Conclusions:** The lack of an adequate serological response to syphilis therapy by delivery among patients treated between 16 and 22 weeks of pregnancy does not appear to be associated with adverse fetal and neonatal outcomes.

## 1. Introduction

Syphilis, caused by *Treponema pallidum*, is a systemic infection and one of the most common sexually transmitted diseases. With approximately 12 million new cases reported annually worldwide, nearly 2 million involve pregnant women [1]. The number of congenital syphilis cases has increased worldwide, depending on the region, as well as the case definitions used, with vertical transmission posing serious risks to fetuses and infants at any stage of pregnancy [2,3,4,5]. Maternal syphilis can lead to adverse pregnancy outcomes, with an associated risk of stillbirth (21%), preterm delivery (6%), and neonatal death (9%) according to WHO estimates in 2016, which also indicated its impact on early fetal deaths and stillbirths as well as preterm or low-birthweight births worldwide [6,7,8]. Hence, the prevalence of syphilis during pregnancy presents a significant public health concern [1,9,10].

The first-line treatment for syphilis is penicillin administered parenterally. The dose and duration of treatment, in turn, depends on the stage of infection. In early syphilis (primary, secondary, or latent), a single dose of benzathine penicillin (2.4 million IU) is recommended, and in late syphilis or syphilis of undetermined duration, three doses (2.4 million IU each) administered at weekly intervals.

The diagnosis of syphilis involves considering clinical symptoms along with confirmation through treponemal blood tests such as TPHA (Treponema pallidum haemagglutination assay) and non-treponemal serological tests like, for example, RPR (rapid plasma reagin) [11,12,13]. It is advisable to report the result of the RPR test in the form of a titer. Non-treponemal reaction titers correlate with disease activity [13,14].

A 4-fold decline in non-treponemal titer between 6 and 12 months after treatment compared to the baseline is considered an adequate serological response [11,12]. So far, no standards have been established for the management of patients who do not have an adequate serological response to treatment [10]. Some experts recommend administering additional doses of penicillin. However, data indicate that additional doses of penicillin do not significantly improve the percentage of patients who achieve an adequate serological response [10,15]. Hence, in patients with an abnormal serological response to treatment, it is recommended that the diagnosis be expanded to include the possible *T. pallidum* involvement of immunologically privileged sites, such as the central nervous system [10,16,17]. Even in 40% of patients with inadequate serological response to syphilis treatment, no central nervous system involvement is found. Such a condition is referred to as serofast syphilis [10,17]. To date, it is unclear whether serofast syphilis is an expression of the persistence of *T. pallidum* in other organs that are inaccessible to testing, such as, for example, the liver or intestinal macrophages, or whether it is simply a form of immune dysregulation associated with the constitutive production of antibodies despite the absence of the pathogen in the body. The ambiguity of this situation is of great diagnostic, epidemiological, and clinical concern [10].

Data on non-HIV infected patients treated for syphilis indicate that a proper serological response to treatment occurs significantly faster than within 6–12 months after the completion of therapy. It is estimated that approximately 70% of patients achieve serological cure as early as the third month post-treatment [18,19]. Serological cure, along with the resolution of clinical symptoms of infection, is considered an indication of bacterial elimination and the non-infectiousness of the treated individual.

So far, it remains uncertain whether patients who do not exhibit a proper serological response are non-infectious. These concerns are particularly significant for female patients treated for syphilis during pregnancy. The gestation period is too short to assess the occurrence of an adequate serological response to treatment six months after its completion. Additionally, it is estimated that up to 60% of pregnant women do not achieve a 4-fold reduction in the RPR reaction titer on the day of delivery compared to the value before the start of treatment, likely due to physiological changes during pregnancy [20,21]. The lack of a decline in the non-treponemal titer or the lack of a proper serological response despite treatment is particularly worrisome in the context of the risk of fetal infection.

The current study aims to assess correlations between the serological response of pregnant women to treatment and fetal condition, newborns’ clinical state, and their serological outcomes.

## 2. Materials and Methods

### 2.1. Characteristics of Patients

Fourteen pregnant patients with early syphilis (stages: secondary or latent) were included in this study, which took place at the Dermatology Outpatient Clinic and the Obstetrics and Gynecology Outpatient Clinic of Jagiellonian University in Cracow, Poland. The diagnosis was based on clinical symptoms, serological blood tests, and medical history, following CDC recommendations [12]. The exclusion criteria comprised chronic inflammatory diseases (including autoimmune diseases), coexisting HIV/HCV/HBV infections, antibiotic use within the past year for any reason, previous treatment for syphilis infection, and unconfirmed duration of less than 1 year for latent-stage infection. All patients provided written consent to participate in the study, which was approved by the Bioethics Committee of Jagiellonian University in Krakow (No. 1072.6120.142.2017, dated 30 November 2017).

### 2.2. Study Plan

All study participants underwent a clinical examination at the time of enrollment, with specific attention given to skin and mucosal changes. Subsequently, venous blood samples were collected for serological tests, including syphilis; for basic blood tests such as complete blood count and CRP (C-reactive protein); and for liver and kidney function tests, as well as for screening for other sexually transmitted diseases.

All patients received a single intramuscular injection of 2.4 million IU benzathine penicillin. Follow-up RPR tests were then performed monthly until delivery.

Based on the results of RPR tests by delivery, patients were stratified into two groups: those showing a 4-fold decline by delivery and those showing no 4-fold decline.

Fetal parameters such as liver length, spleen volume, placental thickness, and peak systolic flow velocity in the middle cerebral artery were assessed via ultrasound every month until delivery for all fetuses.

Newborns received blood tests at birth, including serological tests for syphilis. Additional testing was performed at 3 and 6 months after birth.

### 2.3. Non-Treponemal and Treponemal Tests

RPR reactions were performed at specified time points using the Nadal^®^ RPR Carbon Latex test reagent from Nal von minden, Moers, Germany. The tests followed the manufacturer’s instructions, and titers were consistently read by the same medical laboratory technician.

### 2.4. Statistical Analysis

Statistical analysis was performed using GraphPad Prism 4.0 software (GraphPad Software Inc., San Diego, CA, USA), excluding ROC analysis, for which the medical package of Statistica vs. 13 PL software (StatSoft Inc., Tulsa, OK, USA) was used.

Data for conformity to a normal distribution were tested using the Kolmogorov–Smirnov test. The χ^2^ test (categorical variables) and the Kruskal–Wallis test (continuous variables) were used to evaluate differences between the analyzed groups. In the post hoc analysis, the Dunnet test was used. Correlations between continuous variables were presented as Spearman’s rank-sum coefficient values. A modified Kaplan–Meier estimator was used to demonstrate differences in the rate of decline of RPR titers. A significance level of α < 0.05 was used in each analysis.

## 3. Results

Fourteen pregnant patients aged between 23 and 33, with a median gestational age of 20.5 weeks (range: 16–22 weeks), were diagnosed with early syphilis (in stages: secondary or latent). In eight cases (57.1%), early *T. pallidum* infection was also confirmed in the sexual partner.

Of these pregnant patients, 85.7% were asymptomatic (early latent syphilis), while two had a macular rash on the trunk and one exhibited maculopapular lesions on the soles and palms.

All included patients experienced remission of skin and mucosal lesions within one month after receiving a single intramuscular injection of 2.4 million IU benzathine penicillin without any adverse effects from the treatment.

**Figure 1:**
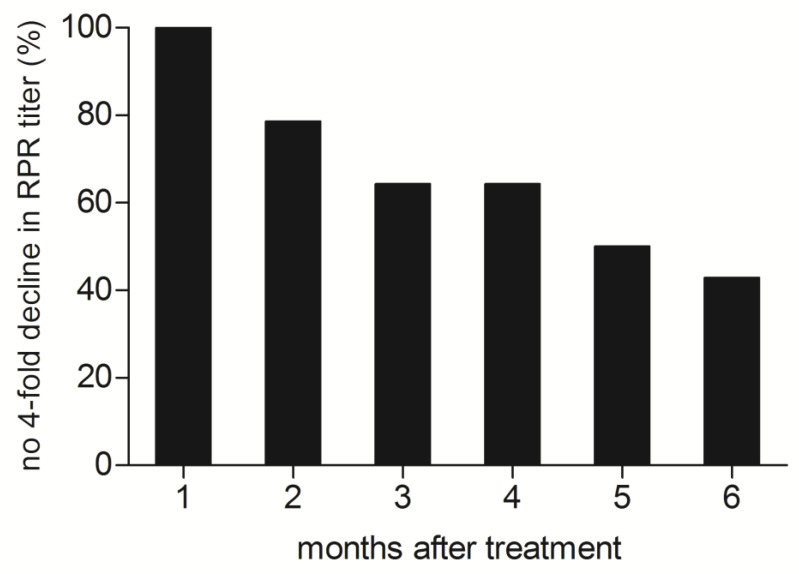
Proportion of pregnant patients without a 4-fold decline in non-treponemal test titers at follow-up after treatment.

In the first month post-treatment, none of the patients showed at least a 4-fold decrease in non-treponemal test titers compared to pre-treatment values; however, by delivery this lack of decrease was observed in half of them (n = 7). Six months after completion of treatment, all patients had already given birth. During the entire study period, none of the patients exhibited an increase in the titer of the non-treponemal test. The proportion of patients showing no 4-fold decline in RPR titers at different follow-up points post-treatment is shown in Figure 1.

Patients from the 4-fold decline in RPR titer by delivery group and the no 4-fold decline group did not differ in basic demographic and clinical characteristics, including age, clinical manifestation of syphilis, and baseline RPR titer (Table 1).

**Figure 2:**
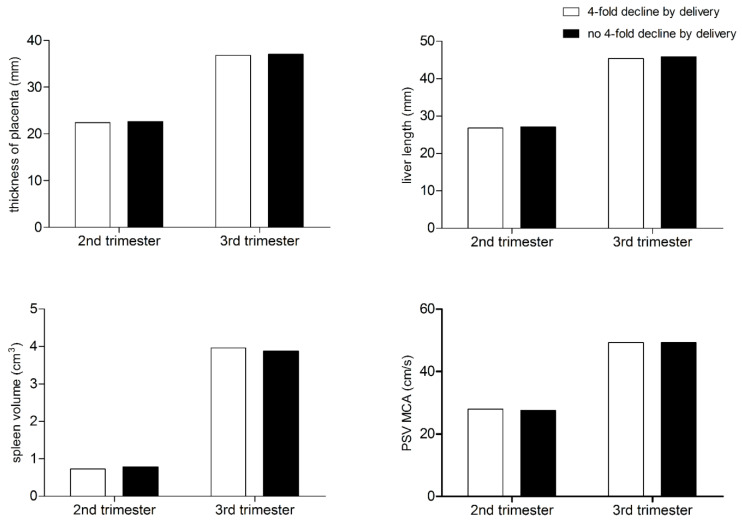
Fetal ultrasound parameters among patients with and without a 4-fold decline in non-treponemal test titers by the time of delivery.

There were also no differences found between the groups with respect to the ultrasound parameters used for fetal assessment (Figure 2).

Additionally, all ultrasonographic findings remained within normal limits for gestational age across all cases, and these findings did not differ between analyzed groups. Detailed fetal parameter values are shown in Table 2.

All pregnancies in this study were delivered via cesarean section. None of the patients experienced significant perioperative complications.

All newborns showed no physical abnormalities or deviations in basic blood tests, including those for anemia and thrombocytopenia, or in elevated liver and kidney function tests, or in active urine sediment tests. None of the newborns’ serum tested positive for IgM-class anti-T. pallidum antibodies.

Initially, all fourteen newborns tested positive for TPHA upon birth; however, only eight children (57.1%) had a positive RPR test. After six-month follow-ups, all the children exhibited seronegativisation for both TPHA and RPR (Table 3).

## 4. Discussion

This study presents data on pregnancy outcomes in individuals undergoing treatment for early syphilis (stages: secondary or latent), comparing those who experienced a 4-fold decrease in RPR titer by delivery with those who did not.

Successful syphilis treatment involves the disappearance of symptoms and a 4-fold decline in non-treponemal titer compared to before treatment. According to CDC guidelines, treatment failure should not be determined until 6–12 months after early-stage syphilis therapy [12]. The lack of a proper serological response can be associated with therapeutic failure and requires further diagnostics, and often also additional treatment. However, it is important to consider that these guidelines are based on studies of nonpregnant individuals, and the normal length of human gestation is insufficient to achieve an adequate serological response.

There have been only a few, mostly retrospective, studies on the serological response to syphilis treatment during pregnancy. They found that over 50% of patients do not achieve a 4-fold decline in RPR titer by delivery [20,21]. These studies identified several factors associated with the 4-fold decline in non-treponemal titer by delivery, including younger maternal age, early-stage syphilis, longer time from treatment to delivery, no history of repeated infections with syphilis, and higher initial RPR titer.

However, it has not yet been established whether the lack of a proper serological response (i.e., at least a 4-fold decline) by delivery, following appropriate treatment of syphilis during pregnancy, can be associated with a risk to the child.

Rac et al. [21], in a retrospective study, found that fewer than half of the women diagnosed with syphilis after 18 weeks of gestation achieved a 4-fold decline in non-treponemal titers by delivery. Interestingly, the risk of having an infant needing treatment for congenital syphilis did not correlate with the 4-fold titer decline by delivery (congenital syphilis rate 20% vs. 16% in no 4-fold decline and 4-fold decline groups, respectively). Our results seem to correspond with those of Rac et al. In our study, a 4-fold decline at least in RPR titer by delivery was not achieved in half of the included women (7 of 14). Despite this, none of the newborns showed clinical or laboratory signs of congenital syphilis at birth or during the six-month follow-up period. The lack of a 4-fold decline in non-treponemal titer at delivery was also not associated with any abnormalities in fetal ultrasound examinations.

In the study by Rac et al. [21], a higher rate of congenital syphilis cases was reported compared to our results, where no cases were found in any child. This difference could be due to the diagnosis and treatment of syphilis being carried out in the late stages of pregnancy. In the Rac et al. study, the average gestational age at treatment was 29 weeks, while in our cohort it was 20.5 weeks. Immediate treatment of the mothers was initiated in each case. Thus, it can be presumed that there has not been an immune response from the fetus to the infection with *Treponema pallidum*, and consequently, no resulting consequences have occurred. Furthermore, our study comprised relatively homogeneous patients with only early-stage syphilis.

Previous studies have identified certain ultrasound abnormalities related to congenital syphilis, including hepatomegaly, placentomegaly, and elevated peak systolic velocity of the middle cerebral artery, with estimated frequencies of 80%, 27%, and 33%, respectively [22,23]. Importantly, these symptoms are primarily associated with the inflammatory response to infection and typically manifest after the 20th week of pregnancy when the fetal immune system matures. Hemolytic anemia is a common abnormality found in fetuses with congenital syphilis. Immune-mediated hemolysis appears to be a significant factor contributing to impaired hematopoiesis, hypersplenism, and nutritional deficiencies linked to impaired placental function. Thus, it has been suggested that the measurement of placental thickness and peak systolic flow velocity in the middle cerebral artery may be useful in the early identification of cases of fetal anemia [24].

In our study, we assessed the above parameters ultrasonographically at monthly intervals until delivery. All measurements fell within the established norms for gestational age. Moreover, we observed no differences in the measurements taken in infants born to mothers with a 4-fold decline in RPR titer by delivery compared to those without an adequate serological response. However, previous data suggest that the absence of fetal ultrasound abnormalities does not exclude congenital syphilis. It has been estimated that up to 12% of newborns with normal ultrasound results may still require penicillin treatment after birth [22,23]. Additionally, ultrasound cannot detect skeletal abnormalities often associated with congenital syphilis. Nonetheless, this procedure appears reasonable considering safety and economic factors.

Since even asymptomatic newborns may exhibit early or late postnatal symptoms, detailed clinical and serological monitoring of newborns and infants born to mothers treated for syphilis during pregnancy is necessary.

Thus, this study additionally presents a prospective assessment of newborns born to women treated for syphilis during pregnancy. The infants underwent clinical and laboratory assessments on the day of delivery and were followed comprehensively for six months. Symptoms such as anemia, thrombocytopenia, elevated serum transaminase activity, active urine sediment, a serum RPR titer in the child’s blood four times higher than in the mother’s, or the presence of anti-T. pallidum IgM antibodies may indicate a diagnosis of congenital syphilis [23]. In the current study, none of the children showed any abnormalities. The next step in evaluating newborns involved analyzing how long it takes for non-treponemal tests in a newborn’s blood serum to become negative. It is hypothesized that if the non-treponemal test remains positive at 6 months of age, there is a high risk of congenital syphilis. In such cases, treatment with penicillin should be initiated even if no other abnormalities are found during physical examination and laboratory tests [25]. Amongst the newborns included in this study, all had reactive treponemal tests on the day of delivery, while 8 out of 14 also tested positive for non-treponemal tests. Interestingly, we observed a significantly higher percentage of newborns testing positive for the non-treponemal test at birth among mothers who did not achieve a 4-fold decline in titers compared to those who did. This suggests that higher maternal antibody levels might result in greater passive transfer to the fetus. By 6 months of age, all infants tested negative for both treponemal and non-treponemal tests. None required treatment, as they were not diagnosed with congenital syphilis. Positive serological findings present at birth were considered passively transmitted from their mothers.

Several study limitations should be acknowledged. Firstly, the small sample size. Despite the increasing incidence of syphilis worldwide, it is a relatively rare disease in developed countries. In 2023, 272,000 children were born in Poland, resulting in a birth rate of 7.2 live births per 1000 inhabitants. This represents a significant decrease compared to 2013, when the rate was 10.3 per 1000 inhabitants. Annually, approximately 2000 patients are diagnosed with syphilis in Poland, of whom only 10% are women. Among these women, about 10% are affected during pregnancy. This indicates that the number of pregnant patients with syphilis is approximately 20 per year. In 2023, based on clinical presentation, laboratory tests, and serological blood tests, congenital syphilis was diagnosed in five children (with an incidence rate of 1.64 per 100,000 inhabitants). However, all patients in our study were diagnosed, treated, and followed up at the same center according to the same protocol. All newborns underwent a full and comprehensive serological and clinical monitoring. Secondly, the study included pregnant patients diagnosed and treated for syphilis between 16 and 22 weeks of gestation. Thus, the results of our study may not be fully applicable to all women who contract syphilis while pregnant, since late prenatal care is an issue in seropositive women. Thirdly, all our patients were diagnosed with early syphilis (stages: secondary or latent); there were no cases of late-stage disease. Nevertheless, it is estimated that the greatest risk to the fetus involves women with early syphilis.

## 5. Conclusions

In summary we have shown that proper treatment for early syphilis (stages: secondary and latent) in pregnant patients between 16 and 22 weeks of pregnancy may result in inadequate serological response for half of them. A lack of 4-fold decline in RPR titer by delivery does not correlate with an increased risk of congenital syphilis in newborns.

Further similar prospective studies are necessary involving larger groups of patients and considering different stages of syphilis as well as various periods of pregnancy when treatment will commence.

## Figures and Tables

**Table 1:** Characteristics of patients included in the study.

	4-Fold Decline Group (n = 7)	No 4-Fold Decline Group (n = 7)	*p*
Age: years (min–max)	27.5 (23–33)	25 (20–29)	0.1
Gestational age at the time of syphilis diagnosis: weeks (min–max)	18 (16–22)	19 (16–22)	0.7
Baseline RPR: titer (min–max)	1:16 (1:8–1:128)	1:32 (1:8–1:128)	0.3
Early latent syphilis: n (%)	5 (71.4)	4 (57.1)	0.2
Macular rash: n (%)	1 (14.3)	1 (14.3)	
Maculopapular rash on palms and soles: n (%)	0	1 (14.3)	0.5
Blood test results at baseline			
Erythrocytes: ×10^6^/uL	5.7 (1.1)	4.8 (1.7)	0.33
Hemoglobin levels: d/dL	14.1 (2.3)	13.7 (3.2)	0.45
Leukocytes: ×10^3^/uL	6.5 (0.5)	5.1 (0.7)	0.08
Lymphocytes: ×10^3^/uL	1.9 (0.3)	1.2 (0.5)	0.75
Neutrophils: ×10^3^/uL	4.2 (0.4)	3.5 (0.2)	0.12
ALT: U/L	37 (4)	32 (2)	0.77
AST: U/L	32 (4.5)	31 (2)	0.6
Creatinine levels: mmol/L	84 (4.3)	74 (8.2)	0.13
CRP levels: mg/dL	11.4 (5.1)	6.1 (2.3)	0.15
Serological characteristics of newborns at the day of delivery			
Positive TPHA: n (%)	7 (100)	7 (100)	
Positive RPR: n (%)	2 (28.6)	6 (85.7)	0.03

Abbreviations: ALT—alanine aminotransferase; AST—aspartate transferase; CRP—c-reactive protein.

**Table 2:** Detailed ultrasound fetal parameter at specific time points.

Fetal Parameters	Gestational Age	4-Fold Decline by Delivery	No 4-Fold Decline by Delivery
Placenta thickness (median: mm)			
	16	21.9	22.3
	20	22.4	22.6
	24	27.1	26.9
	28	33.8	33.6
	32	34.1	34.2
	36	39.5	39.8
	40	40.2	40.4
Liver length (median: mm)			
	16	19.1	19.3
	20	26.8	27.1
	24	31.1	31
	28	33.5	33.8
	32	42.1	42.7
	36	48.6	49.1
	40	55.1	54.3
Spleen volume (median: cm^3^)			
	16	0.67	0.62
	20	0.73	0.79
	24	1.16	1.35
	28	2.28	2.45
	32	3.96	3.88
	36	7.8	7.2
PSV MCA (median: cm/s)			
	20	25.6	26.1
	24	30.5	29.1
	28	36.9	36.1
	32	45.1	44.8
	36	53.5	53.9
	40	64.8	65.1

Abbreviations: PSV MCA—peak systolic velocity in middle cerebral artery.

**Table 3:** Results of serological blood tests for syphilis (RPR, TPHA) in children of mothers with syphilis, at specific time points.

		Day of Delivery		Third Month of Life		Sixth Month of Life	
Child	Sex	RPR	TPHA	RPR	TPHA	RPR	TPHA
Neonate 1	Female	1:2	positive	negative	negative	negative	negative
Neonate 2	Female	negative	positive	negative	negative	negative	negative
Neonate 3	Female	1:2	positive	negative	negative	negative	negative
Neonate 4	Female	1:4	positive	1:2	positive	negative	negative
Neonate 5	Female	negative	positive	negative	negative	negative	negative
Neonate 6	Female	negative	positive	negative	negative	negative	negative
Neonate 7	Female	1:4	positive	negative	negative	negative	negative
Neonate 8	Female	1:4	positive	1:2	positive	negative	negative
Neonate 9	Male	negative	positive	negative	negative	negative	negative
Neonate 10	Male	1:4	positive	1:2	positive	negative	negative
Neonate 11	Male	negative	positive	negative	positive	negative	negative
Neonate 12	Male	negative	positive	negative	positive	negative	negative
Neonate 13	Male	1:4	positive	1:2	positive	negative	negative
Neonate 14	Male	1:4	positive	1:2	positive	negative	negative

## Data Availability

The raw data supporting the conclusions of this article will be made available by the authors on request.

## References

[1] Kojima N., Klausner J.D. (2018). An Update on the Global Epidemiology of Syphilis. Curr. Epidemiol. Rep..

[2] Gilmour L.S., Walls T. (2023). Congenital Syphilis: A Review of Global Epidemiology. Clin. Microbiol. Rev..

[3] Adhikari E.H. (2020). Syphilis in Pregnancy. Obstet. Gynecol..

[4] Bowen V., Su J., Torrone E., Kidd S., Weinstock H. (2015). Increase in incidence of congenital syphilis-United States, 2012–2014. MMWR Morb. Mortal. Wkly. Rep..

[5] Torrone E.A., Miller W.C. (2018). Congenital and Heterosexual Syphilis: Still Part of the Problem. Sex. Transm. Dis..

[6] Gomez G.B., Kamb M.L., Newman L.M., Mark J., Broutet N., Hawkes S.J. (2013). Untreated maternal syphilis and adverse outcomes of pregnancy: A systematic review and meta-analysis. Bull. World Health Organ..

[7] D’Aiuto C., Valderrama A., Byrns M., Boucoiran I. (2020). Sexually Transmitted and Blood-Borne Infections in Pregnant Women and Adverse Pregnancy Outcomes. J. Obstet. Gynaecol. Can..

[8] Gulersen M., Lenchner E., Eliner Y., Grunebaum A., Johnson L., Chervenak F.A., Bornstein E. (2023). Risk factors and adverse outcomes associated with syphilis infection during pregnancy. Am. J. Obstet. Gynecol. MFM.

[9] Eppes C.S., Stafford I., Rac M. (2022). Syphilis in pregnancy: An ongoing public health threat. Am. J. Obstet. Gynecol..

[10] Cao Q., Li Y., Hu Y., He B., Tang Y., Cao T., Peng B., Zhou X., Liu S. (2024). Serofast status in syphilis: Pathogenesis to therapeutics. Clin. Chim. Acta.

[11] Janier M., Unemo M., Dupin N., Tiplica G.S., Potočnik M., Patel R. (2021). 2020 European guideline on the management of syphilis. J. Eur. Acad. Dermatol. Venereol..

[12] Centers for Disease Control and Prevenation (2021). Sexually Transmitted Diseases Treatment Guidelines 2021.

[13] Satyaputra F., Hendry S., Braddick M., Sivabalan P., Norton R. (2021). The Laboratory Diagnosis of Syphilis. J. Clin. Microbiol..

[14] Pillay A. (2018). Centers for Disease Control and Prevention Syphilis Summit-Diagnostics and Laboratory Issues. Sex. Transm. Dis..

[15] Liu Y., Bian Q., Zhang S., Wang J., Wang Z., Li J. (2020). Is repeated retreatment necessary for HIV-negative serofast early syphilis patients?. Exp. Ther. Med..

[16] Lafond R.E., Lukehart S.A. (2006). Biological basis for syphilis. Clin. Microbiol. Rev..

[17] Seña A.C., Zhang X.H., Li T., Zheng H.P., Yang B., Yang L.G., Salazar J.C., Cohen M.S., Moody M.A., Radolf J.D. (2015). A systematic review of syphilis serological treatment outcomes in HIV-infected and HIV-uninfected persons: Rethinking the significance of serological non-responsiveness and the serofast state after therapy. BMC Infect. Dis..

[18] Luo Z., Ding Y., Yuan J., Tian L., Zhang L., Wu Q., Mou J. (2021). Predictors of serological cure after penicillin therapy in HIV-negative patients with early syphilis in Shenzhen, China. PLoS ONE.

[19] Pastuszczak M., Gozdzialska A., Jakiela B., Obtulowicz A., Jaskiewicz J., Wojas-Pelc A. (2017). Robust pro-inflammatory immune response is associated with serological cure in patients with syphilis: An observational study. Sex. Transm. Infect..

[20] Galan H.L., Montalvo J.F., Deaver J. (1997). Retrospective analysis of the serologic response to the treatment of syphilis during pregnancy. Infect. Dis. Obstet. Gynecol..

[21] Rac M.W., Bryant S.N., Cantey J.B., McIntire D.D., Wendel G.D., Sheffield J.S. (2015). Maternal titers after adequate syphilotherapy during pregnancy. Clin. Infect. Dis..

[22] Rac M.W., Bryant S.N., McIntire D.D., Cantey J.B., Twickler D.M., Wendel G.D., Sheffield J.S. (2014). Progression of ultrasound findings of fetal syphilis after maternal treatment. Am. J. Obstet. Gynecol..

[23] Hollier L.M., Harstad T.W., Sanchez P.J., Twickler D.M., Wendel G.D. (2001). Fetal syphilis: Clinical and laboratory characteristics. Obstet. Gynecol..

[24] Ahmed M., Jackson D.E. (2024). The role of measuring peak systolic velocity of the middle cerebral artery blood flow and anti-K1 titre during pregnancy to detect foetuses with severe anaemia, foetal hydrops, and the requirement of intrauterine transfusion: A systematic review and meta-analysis. Hematol. Transfus. Cell Ther.

[25] Pastuszczak M., Wojas-Pelc A. (2013). Current standards for diagnosis and treatment of syphilis: Selection of some practical issues, based on the European (IUSTI) and U.S. (CDC) guidelines. Postepy Dermatol. Alergol..

